# Research Progress on the Quorum Sensing System of *Acinetobacter baumannii* and Its Inhibitors

**DOI:** 10.3390/pathogens15070731

**Published:** 2026-07-13

**Authors:** Jing Liao, Xingxin Liu, Jingjing Luo, Jiaji Ling, Liting Liang, Ziyi Yan, Wenjing Wu, Wei Zhou, Yongmei Jiang

**Affiliations:** 1 Department of Laboratory Medicine, West China Second University Hospital, Sichuan University, Chengdu 610041, China; xiaoliaojing@hotmail.com (J.L.);; 2 Key Laboratory of Birth Defects and Related Diseases of Women and Children (Sichuan University), Ministry of Education, Chengdu 610041, China

**Keywords:** *Acinetobacter baumannii*, quorum sensing system, resistance mechanisms, inhibitors, quorum quenching

## Abstract

The escalating problem of bacterial drug resistance poses a severe threat to global public health, with *Acinetobacter baumannii* (*A. baumannii*) exhibiting particularly high resistance rates that are closely linked to its Quorum Sensing (QS) system. This narrative review synthesizes current knowledge on the *A. baumannii* QS system, focusing on its essential role in mediating antimicrobial resistance, as well as the latest progress in developing QS inhibitors. Key findings indicate that the *A. baumannii* QS system enhances bacterial survival by promoting biofilm formation, and regulating the expression of efflux pump and resistance genes. However, significant translational challenges remain, including the risk of inducing resistance, the poor bioavailability and suboptimal pharmacokinetic properties, and their reduced efficacy in complex biological systems. In conclusion, while QS inhibitors represent a promising therapeutic target, overcoming current developmental bottlenecks requires combining them with traditional antibiotics, exploring novel drug delivery strategies, and integrating cutting-edge tools to accelerate drug discovery and clinical application.

## 1. Introduction

*Acinetobacter baumannii* (*A. baumannii*), a Gram-negative bacillus, is a formidable opportunistic pathogen responsible for a wide range of localized and systemic infections, including pneumonia, bacteremia, meningitis, endocarditis, and urinary tract and skin infections. Classified as a Category 3 human-transmissible pathogen, it has become a major source of nosocomial infections. Data from the China Antimicrobial Surveillance Network (CHINET) for 2025 indicate that the detection rate of *A. baumannii* among all clinical isolates was 7.1%, ranking fifth [1]. A study in The Lancet Infectious Diseases reported that *A. baumannii* infections contribute to 16.7 million disability-adjusted life years (DALYs) worldwide, causing an average loss of 215.8 healthy life years per 100,000 population [2]. Concurrently, the increase in antimicrobial resistance in *A. baumannii* has severely limited therapeutic options, leading to higher patient mortality, prolonged hospital stays, and increased healthcare costs [3]. Data from CHINET (2005–2025) reveal a sustained upward trend in resistance rates to multiple commonly used antibiotics [4]. A 2022 report in The Lancet estimated that high-level resistance in *A. baumannii* is responsible for approximately 400,000 deaths globally each year [5], with carbapenem resistance rates exceeding 50% in clinical isolates from several regions. Consequently, *A. baumannii* is recognized as a major threat to global healthcare systems [6,7]. Given its resistance to most available antibiotics and its association with high morbidity and mortality, there is an urgent need for new alternative strategies to treat *A. baumannii* infections. In 2024, the World Health Organization (WHO) re-listed carbapenem-resistant *A. baumannii* as a “critical” priority pathogen on its list of bacteria for which new antibiotics are urgently needed [8].

Recent research has highlighted the quorum sensing (QS) system not only as a key regulator of bacterial resistance but also as a promising therapeutic target. The QS system is a chemical communication mechanism that bacteria use to coordinate group behaviors. By monitoring the concentration of secreted signal molecules, bacteria can track changes in their population density. When the signal concentration reaches a specific threshold, it binds to a transcriptional receptor protein, triggering the expression of downstream genes that modulate biological behaviors such as increased drug resistance, virulence factor synthesis, and biofilm formation [9,10]. The inhibition of the QS system, known as quorum quenching (QQ), is considered a potential anti-microbial strategy. It can reduce pathogen adhesion and virulence, and enhance bacterial susceptibility to antibiotics [11]. Since inhibiting the QS system does not directly kill bacteria, the selection pressure for developing resistance is significantly lower. This review summarizes the components of the *A. baumannii* QS system, its mechanisms for regulating resistance, and the current progress in developing QS inhibitors, aiming to elucidate their research status and explore their potential applications.

## 2. QS Systems in Acinetobacter Species

### 2.1. The QS System in A. baumannii

The QS system in Gram-negative bacteria typically comprises three main components: signal molecules, signal synthases, and transcriptional receptors [12]. The primary signal molecules are N-acyl-homoserine lactones (AHLs), which are synthesized from S-adenosyl methionine (SAM) and various fatty acyl-acyl carrier proteins (acyl-ACPs), resulting in acyl chains of varying lengths (C_4_–C_18_). These AHLs are classified as short-chain or long-chain and can have different substituents at the C-3 position, such as hydroxyl, oxo, or methylene groups [13,14]. *A. baumannii* predominantly secretes long-chain AHLs, with N-(3-hydroxydodecanoyl)-L-homoserine lactone (3-OH-C_12_-HSL) being the most abundant [15,16]. Table 1 summarizes the types of AHL signal molecules secreted by *A. baumannii*. The key genes of the *A. baumannii* QS system are *abaI* and *abaR*, which are homologous to the *luxI* and *luxR* genes of *Vibrio fischeri* (*V. fischeri*) [16]. The *abaI* gene encodes the AbaI protein, a signal synthase, which is currently the only known AHL synthase in *A. baumannii*. The *abaR* gene encodes the AbaR protein, an intracellular receptor for AHLs. Upon binding to AHLs, the AbaR-AHL complex acts as a transcriptional regulator, binding to the promoter region of target DNA to initiate a cascade of gene expression. Whole-genome sequencing of *A. baumannii* has identified a putative palindromic sequence (CTGTAAATTCTTACAG), locating 59 nucleotides upstream of the *abaI* start codon, which is predicted to be the AbaR binding site, often referred to as the “lux box” [17].

### 2.2. QS Systems in Other Acinetobacter Species

Similar QS systems have been identified in other species of the *Acinetobacter* genus. In *Acinetobacter nosocomialis* (*A. nosocomialis*), an AnoI/AnoR-regulated QS system has been characterized. AnoI/AnoR are homologous to AbaI/AbaR, with both pairs sharing a 91% sequence identity. *A. nosocomialis* also secretes the 3-OH-C_12_-HSL signal molecule, and its AnoI/AnoR system has been shown to regulate surface motility and play a crucial role in biofilm formation [18]. In *Acinetobacter* strains DR1 and GG2, the LuxI homolog AqsI and the synthase gene *aciI* have been identified, respectively. Deletion of *aqsI* impaired biofilm formation, which could be restored by the addition of exogenous signal molecules [19]. GG2 strain produces two long-chain signal molecules: 3-OH-C_12_-HSL and N-(3-oxododecanoyl)-L-homoserine lactone (3-oxo-C_12_-HSL) [20].

## 3. QS System-Mediated Resistance Mechanisms in *A. baumannii*

The resistance mechanisms of *A. baumannii* are complex, involving changes in outer membrane permeability [21], enhanced efflux pump activity [22], production of antibiotic-inactivating enzymes [23,24], and target site modifications [23]. The QS system contributes to this resistance through several pathways. First, the regulation of biofilm formation is a key QS system-mediated resistance pathway. Components such as extracellular polysaccharides, proteins, and nucleic acids constitute a relatively dense biofilm matrix that encloses the bacteria. This matrix acts as a physical barrier, impeding the penetration of antibiotics. Furthermore, bacteria within biofilms can enter a dormant-like state, characterized by reduced metabolic activity and decreased uptake of antimicrobial agents. Multiple studies have confirmed that the QS system promotes biofilm formation in *A. baumannii*. Researchers such as Niu et al. [25] and Chow et al. [26] constructed *abaI* gene knockout strains and observed a significant reduction in biofilm mass compared with the wild-type. This biofilm-forming ability was restored upon the addition of exogenous AHLs, demonstrating that the loss of the signal synthase gene inhibits biofilm formation. The QS system also regulates genes associated with biofilm formation and surfactant synthesis [27]. Studies in *Pseudomonas aeruginosa* (*P. aeruginosa*) have shown that QS inactivation leads to structurally simplified biofilms. Mature biofilms are typically highly resistant to environmental stressors like UV radiation and antibiotics [28,29,30], whereas biofilms formed by QS-deficient mutants are more susceptible.

The QS system is also connected with the expression of efflux pump-related genes, and the two exhibit mutual influence. The regulation of Resistance-Nodulation-Division (RND) family efflux pumps by QS was first discovered in *P. aeruginosa*, where AHLs were found to induce the expression of the MexGHI-OpmD efflux pump system [31]. Similar regulatory links have been observed in other bacteria, including *Burkholderia glumae* (*B. glumae*), *Bacteroides fragilis* (*B. fragilis*), and *Burkholderia pseudomallei* (*B. pseudomallei*) [32,33,34]. In *A. baumannii*, the QS system has been shown to regulate the expression of RND family efflux pump genes. Compared with the wild-type, an *abaI* knockout mutant exhibited significantly lower expression levels of *adeA* and *adeB*, which were restored by adding AHLs [9]. The AdeABC efflux pump is a major contributor to resistance against β-lactams, aminoglycosides, quinolones, and tetracyclines [35,36,37,38], and upregulation of *adeB* is associated with the emergence of pandrug-resistant *A. baumannii* [39]. Subhadra et al. [40] also found that in *A. nosocomialis*, the QS system and the RND-family efflux pump AcrAB exert mutual influences. Deletion of the anoR gene leads to the downregulation of acrAB expression, while the AcrR protein can also regulate the expression of QS system-related genes by binding to the promoter regions of anoI and anoR [40]. Deletion of acrR results in reduced signal molecule secretion, indicating a complex feedback loop. Finally, regulation of the expression of resistance genes is also one of the QS-mediated mechanisms of antibiotic resistance. Research has shown that the AbaI/AbaR system can directly or indirectly regulate the expression of specific resistance genes [41]. For instance, it can upregulate the expression of *bla*_OXA-51_ and *ampC* genes, thereby increasing resistance of strains to meropenem [9]. This demonstrates that the QS system enhances antibiotic resistance in *A. baumannii* through multiple, coordinated mechanisms.

## 4. Current Status of QS System Inhibitor Research and Development

Given the extensive regulatory role of the QS system in bacterial resistance and virulence, it represents a highly promising target for the development of novel antimicrobial agents [42]. QS inhibitors can be used in combination with traditional antibiotics to enhance their efficacy, reduce required dosages, and slow the development of resistance. Since inhibition of the QS system does not exert a direct bactericidal effect, bacteria are less likely to develop resistance against it. This approach has already been a major strategy in the development of new drugs against several bacterial pathogens [42]. Furthermore, some natural QS inhibitors have already entered clinical trials and demonstrated promising results. For instance, β-caryophyllene (a terpenoid) has shown favorable clinical efficacy in the treatment of *Helicobacter pylori* infection, resulting in the improvement of epigastralgia and nausea, as well as decreased serum IL-1β levels [43]. In addition, baicalein has been shown to exert a dose-dependent antibiofilm effect against *Streptococcus mutans* (the main cariogenic pathogen), leading to a significant decrease in the total biomass area [44].

There are three primary strategies for inhibiting QS: (1) reducing the expression or impairing the function of the signal synthase; (2) degrading the signal molecules; and (3) reducing the expression of the receptor protein or using competitive antagonists to block signal–receptor binding [45]. The three categories of inhibitors and their mechanisms of action are summarized in Table 2.

### 4.1. Targeting Signal Synthase to Inhibit QS System

**Figure 1:**
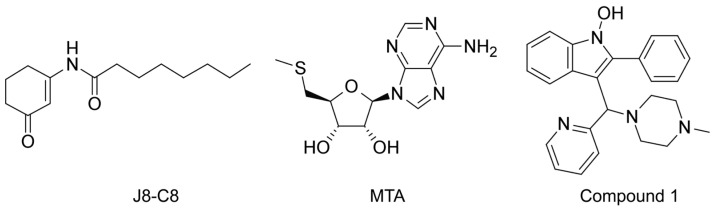
Chemical structures of representative AHLs synthase inhibitors. The figure illustrates three notable inhibitors targeting AHL synthases: J8-C8, a signal molecule analog that inhibits the synthase by occupying the acyl-chain binding site; MTA (5′-methylthioadenosine), a reaction byproduct that binds to the SAM substrate site; and Compound 1, a potent inhibitor identified through enzyme-coupled high-throughput screening methods.

Enzyme-coupled high-throughput screening methods have been developed to discover inhibitors of AHL synthases [46]. Using this approach, several potent inhibitors have been identified, such as Compound 1. It is also hypothesized that the activity of AHL synthases of Gram-negative bacteria can be inhibited by substrate analogs, such as S-adenosylhomocysteine, or reaction byproducts like 5′-methylthioadenosine (MTA). A notable synthase inhibitor, J8-C8, was identified from a library of signal molecule analogs and reported in PNAS [47]. X-ray crystallography revealed that J8-C8 binds to the TofI synthase of *B. glumae* by occupying the acyl-chain binding site of the acyl-ACP substrate. The byproduct MTA was also found to independently bind to the SAM substrate site. Further research on them could facilitate the development of targeted synthase inhibitors. Their chemical structures are presented in Figure 1.

### 4.2. Targeting Signal Molecules to Inhibit QS System

The QS system can be inhibited by the enzymatic degradation of signal molecules using QQ enzymes [26]. Since AHLs consist of a homoserine lactone ring and an acyl side chain, QQ enzymes are classified into two main types: lactonases, which target the lactone ring, and acylases, which act on the acyl chain. These enzymes can be endogenous, produced by the bacterium itself for self-regulation, or exogenous, produced by other microorganisms in the environment. A single QQ enzyme can often recognize multiple types of AHLs, and a specific AHL can also be hydrolyzed by different QQ enzymes [16]. In *A. baumannii*, a novel QQ enzyme, AidA, an α/β hydrolase, was discovered to be induced by the external addition of 3-oxo-C_12_-HSL. AidA hydrolyzes signal molecules, leading to reduced motility and biofilm formation [48]. Another study found that, in addition to AidA, three lactonase sequences were also identified in the *A. baumannii* genome [16]. Exogenous QQ enzymes can also degrade *A. baumannii* signals. For example, MomL from *Muricauda olearia* (*M. olearia*) and Aii20J from a marine bacterium can both degrade AHLs produced by *A. baumannii* [16,49]. MomL, a lactonase, not only reduces biofilm formation but also increases the susceptibility of *A. baumannii* to multiple antibiotics [50]. Aii20J also reduces motility and biofilm formation. Additionally, ethyl acetate extracts from plants like *Thladiantha dubia* (*T. dubia*) and *Rubia cordifolia* (*R. cordifolia*) have been shown to inhibit AHL synthesis in *A. baumannii*, although the specific mechanisms are not yet clear.

### 4.3. Targeting Signal Receptors to Inhibit QS

**Figure 2:**
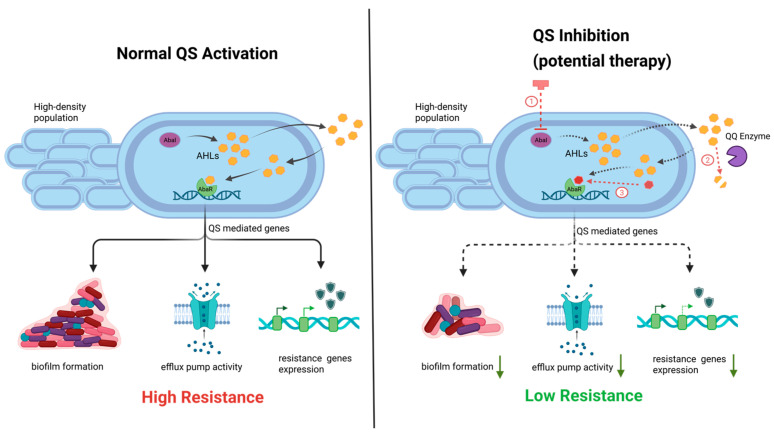
QS system-mediated mechanisms and targeted inhibition strategies in *A. baumannii*. (**Left**): Normal QS activation. The AbaI synthase produces AHLs. Upon reaching a threshold, AHLs bind to the AbaR receptor, inducing a cascade of reactions, including increasing biofilm formation, efflux pump activity, and resistance genes expression, that enhance antimicrobial resistance. (**Right**): Three QS inhibition strategies. ① Inhibiting the AbaI synthase to block AHL production. ② Degrading secreted AHLs by quorum quenching (QQ) enzymes. ③ Disrupting the function of the AbaR or utilizing signal molecule analogs to hinder their binding. These interventions effectively suppress resistance pathways, leading to low bacterial resistance. Created in BioRender. Dao Tu. (2026) https://BioRender.com/kynk5zz (accessed on 29 June 2026).

Targeting signal receptors can be achieved in two ways. The first is to interfere with the expression or translation of the receptor protein or disrupt its ligand-binding domain and catalytic active site. Nicol et al. [51] identified specific unsaturated fatty acids, such as myristoleic acid and palmitoleic acid, that significantly inhibit the transcription of the *abaR* gene, thereby reducing AbaR protein levels. At sub-inhibitory concentrations, these fatty acids markedly reduced motility and biofilm formation in *A. baumannii* ATCC 17978 [51]. The second approach involves using natural or synthetic analogs of signal molecules to competitively block the receptor’s binding site, preventing it from functioning as a transcription factor, thereby interfering with the expression of downstream target genes [52,53,54,55,56]. Examples include furanone C-30, thiazolidinedione derivatives, and dioxazaborocanes, which have shown inhibitory effects on LuxR family proteins [57,58,59,60,61,62,63]. Alkylamine-modified cyclodextrins have also been shown to inhibit the QS system by altering the conformation of these molecules [64]. Swem et al. [65] demonstrated that the small molecule 4606–4237 is a potent antagonist of the CviR receptor, and its optimized derivatives showed even stronger activity, significantly reducing QS-mediated pathogenicity. Screening of a signal molecule analog library also identified E9C-3oxoC6, an inhibitor that competitively blocks signal binding to the TofR protein in *B. glumae* [47]. Figure 2 clearly illustrates the three inhibition strategies and their regulatory pathways.

## 5. Challenges in the Development of QS Inhibitors

Inhibition of the QS system may still induce the development of bacterial resistance [66,67], which is a potential risk that warrants close attention during the development of QS inhibitors. Moreover, the underlying resistance mechanisms may involve multiple aspects, posing additional challenges to addressing this issue. Previous studies have reported that certain opportunistic pathogens are already capable of developing resistance to QS inhibitors. For instance, bacteria may upregulate efflux pumps to expel inhibitors from the cell, as observed in *P. aeruginosa*, which developed resistance to furanone C-30 through mutations leading to increased efflux [67]. Physiological adaptation can also lead to resistance. QS inhibitors may increase the selective pressure experienced by bacteria, under which inhibition of the QS system may in turn drive bacteria to further enhance their QS functionality. Ultimately, this adaptation could counteract the effects of QS inhibitors [68]. Furthermore, oxidative stress can select for bacteria with highly active QS systems, correspondingly, these strains also exhibit stronger resistance to oxidative stress [68]. This may lead to a reduction in the intracellular levels of QS inhibitors, ultimately resulting in enhanced virulence.

Many natural QS inhibitors exhibit poor bioavailability and suboptimal pharmacokinetic properties [69]. For example, polyphenols such as quercetin and curcumin possess limited aqueous solubility, which compromises their bioavailability in systemic circulation [70]. Similarly, compounds like brominated furanones and resveratrol are susceptible to rapid degradation or hepatic enzymatic metabolism under physiological conditions, resulting in short half-lives and diminished systemic therapeutic efficacy [71,72].

Lastly, the vast majority of current QS inhibitors are supported solely by in vitro data. Their efficacy, safety, and specificity remain to be rigorously validated within complex biological systems. However, owing to discrepancies in bacterial physiology and host factors, some inhibitors fail to replicate their in vitro success in animal models. More importantly, disrupting bacterial communication networks could potentially trigger unintended adverse effects on the host microbiome [73].

## 6. Future Trends and Prospects

The combination of QS inhibitors with traditional antibiotics is a promising strategy for treating drug-resistant infections. This approach has demonstrated significant synergistic effects, enhancing antibacterial efficacy and providing a novel strategy for clinical treatment [74,75,76]. For example, ajoene, furanone C-30, and horseradish extract have been shown to potentiate the activity of tobramycin against resistant *P. aeruginosa* [77,78,79]. Curcumin has shown synergy with gentamicin and azithromycin, as well as with ceftazidime and ciprofloxacin [80], allowing for a reduction in antibiotic dosage to as low as 1/16 of the minimum inhibitory concentration (MIC) [81]. Tobramycin, colistin, and ciprofloxacin, when used in combination with N-(2-pyrimidinyl)butylamine, can also enhance their antibacterial activity against *P. aeruginosa* [82]. These findings confirm that combination therapy has excellent potential, enabling lower antibiotic doses and reducing the risk of resistance. However, it will be necessary to further investigate the combination regimens and potential side effects, in order to fully exploit the advantages of combining QS inhibitors with traditional antimicrobials and to provide more effective therapeutic strategies for addressing bacterial resistance.

To address the pharmacokinetic shortcomings, drug delivery strategies can be further explored. For example, a recent article published in ACS Nano demonstrated that a quercetin-loaded star-shaped antibacterial peptide polymer, developed based on a “drug-in-drug” strategy, can eliminate over 99.99% of biofilms [83]. Additionally, it exhibited outstanding performance in relieving ocular inflammation, ultimately contributing to the restoration of visual function. In addition, the development of synthetic inhibitors also warrants more attention. Synthetic compounds offer advantages such as precise targeting, well-defined structures, and ease of modification and optimization. Structure-based drug design, using techniques like cryo-electron microscopy to resolve the structures of key QS proteins, can provide detailed information for inhibitor design. Molecular docking can be used to screen large compound libraries computationally. Looking ahead, artificial intelligence (AI) approaches will hold tremendous potential for advancing inhibitor discovery and structure-based drug design. For instance, when the structure of a target protein remains unavailable, advanced deep-learning algorithms such as AlphaFold can be employed to predict its 3D architecture. Meanwhile, AI platforms like DrugCLIP enable ultra-high-throughput virtual screening of compound libraries, which not only significantly accelerates the screening process but also substantially reduces associated experimental costs. Increased research in these areas will be vital for advancing the development of effective QS inhibitors.

## 7. Conclusions

The rising resistance of *A. baumannii* presents a formidable clinical challenge, and the QS system has emerged as a promising new target for therapeutic intervention. *A. baumannii* primarily secretes long-chain signaling molecules dominated by 3-OH-C_12_-HSL, which are synthesized by the AbaI protein and subsequently regulate the expression of target genes through binding to the AbaR protein. The QS system regulates resistance through multiple mechanisms, including promoting biofilm formation, upregulating efflux pumps, and controlling the expression of resistance genes like *bla*_OXA-51_ and *ampC*.

Significant progress has been made in the development of QS system inhibitors, which target the synthase, the signal molecule, or the receptor. Inhibitors such as J8-C8, QQ enzymes like AidA and MomL, and receptor antagonists like unsaturated fatty acids and furanone C-30 have shown promising activity. However, their research still faces multiple challenges, including the non-negligible risk of inducing drug resistance, the poor bioavailability and suboptimal pharmacokinetic properties of many natural QS inhibitors, and the reduced efficacy of some inhibitors in complex biological systems. The combination of QS inhibitors with traditional antibiotics is one of the key application strategies. Further studies on combination regimens and potential side effects are needed to fully maximize their advantages. Furthermore, exploring novel drug delivery strategies and integrating interdisciplinary approaches to continuously innovate research methods, including the application of cutting-edge AI tools, will greatly accelerate inhibitor development. A breakthrough in this field will provide much-needed effective treatments for infections caused by this critical pathogen.

## Figures and Tables

**Table 1:** Types of AHLs produced by *Acinetobacter baumannii*.

Synonyms	Chemical Name	Chemical Structure
C_10_-HSL	N-Decanoyl-L-homoserine lactone	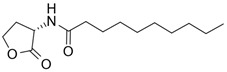
C_12_-HSL	N-Dodecanoyl-L-homoserine lactone	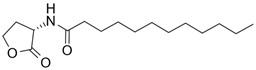
3-OH-C_12_-HSL	N-(3-Hydroxydodecanoyl)-L-homoserine lactone	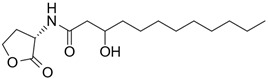
3-oxo-C_13_-HSL	N-(3-Oxo-tridecanoyl)-L-homoserine lactone	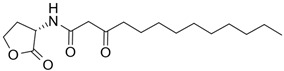
C_14_-HSL	N-Tetradecanoyl-L-homoserine lactone	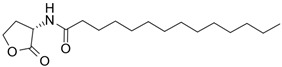
C_16_-HSL	N-Hexadecanoyl-L-homoserine lactone	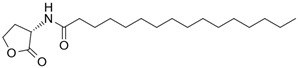

**Table 2:** Summary of the three categories of QS system inhibitors.

Inhibitor Category	Mechanism of Action	Representative Inhibitors	Key Effects
1. Targeting signal synthase	Reduces the expression of synthase genes or impairs the function of the synthase enzyme to block AHL production.	J8-C8	A small molecule that occupies the acyl-chain binding site.
		MTA	A reaction byproduct that binds to the SAM substrate site.
		Compound 1	Not clear.
2. Targeting signal molecules	Uses QQ enzymes to degrade AHL signals.	AidA MomL Aii20J	Lactonases, which target the lactone ring, or acylases, which act on the acyl chain.
		Ethyl acetate extracts	Not clear.
3. Targeting signal receptors	Interferes with receptor protein expression or uses competitive antagonists to block the ligand-binding domain.	Unsaturated fatty acids	Repress the transcription of signal receptor genes.
		Furanone C-30, thiazolidinedione derivatives, dioxazaborocanes, alkylamine-modified cyclodextrins, small molecule 4606–4237, E9C-3oxoC6.	Competitive antagonists that block the receptor’s binding site.

## Data Availability

No new data are included in this article. Data in referenced prior studies may be accessible in the original works.
